# Severe Gamma-Hydroxybutyrate (GHB) Withdrawal: A Case Report and Clinical Management

**DOI:** 10.1192/j.eurpsy.2026.10836

**Published:** 2026-07-31

**Authors:** I. M. Peso Navarro, C. Garcia Cerdan, M. Ligero Argudo, M. Bersabe Perez, M. Covacho Gonzalez, C. Munaiz Cossio

**Affiliations:** 1 Psiquiatria, Complejo Asistencial de Salamanca; 2 Psiquiatria, Complejo Asistencial Salamanca, Salamanca; 3 Psicología, Hospital Gregorio Marañon, Madrid, Spain

## Abstract

**Introduction:**

Gamma-hydroxybutyrate (GHB) is a central nervous system depressant with high abuse potential, often used recreationally for its euphoric and disinhibitory effects. Chronic use may lead to tolerance, dependence, and a severe withdrawal syndrome, which can resemble alcohol or benzodiazepine withdrawal but often presents with faster onset and greater medical risk. Early recognition and appropriate management are crucial.

**Objectives:**

To describe a case of GHB dependence and acute withdrawal, highlighting diagnostic challenges, clinical course, and therapeutic interventions.

**Methods:**

Case report of a young adult with a history of recreational GHB use, admitted to the psychiatric emergency unit due to agitation, confusion, and autonomic symptoms after abrupt cessation. Clinical data were obtained through direct assessment, collateral history, and medical chart review. Treatment strategies included pharmacological stabilization, monitoring of vital signs, and supportive interventions.

**Results:**

The patient presented with acute withdrawal symptoms within hours of last GHB intake, including tremor, diaphoresis, hypertension, agitation, anxiety, and episodes of disorientation. High-dose benzodiazepines were required for symptom control, with gradual clinical stabilization over several days. Adjunctive medications were necessary to address residual insomnia and anxiety. Psychoeducation regarding relapse risk and long-term consequences of GHB use was provided, and referral to an addiction treatment program was arranged.

**Conclusions:**

GHB withdrawal constitutes a psychiatric and medical emergency that can mimic other withdrawal syndromes but requires rapid recognition and aggressive benzodiazepine-based management. Long-term care should combine pharmacological strategies for acute stabilization with psychosocial interventions to reduce relapse risk. This case underscores the importance of awareness among clinicians due to increasing prevalence of GHB use in nightlife and high-risk populations.

**Disclosure of Interest:**

None Declared

